# Supplementary material for: The Effect of Necrosis Inhibitor on Dextran Sulfate Sodium Induced Chronic Colitis Model in Mice
Source: Pharmaceutics. 2023 Jan 9;15(1):222. doi: 10.3390/pharmaceutics15010222 (PMC9862178; doi:10.3390/pharmaceutics15010222)
Supplement: Supplementary file 1 [file pharmaceutics-15-00222-s001.zip › pharmaceutics-2102662-supplementary.pdf]

pharmaceutics

MDPI

Supplementary Materials: The Effect of Necrosis Inhibitor on Dextran Sulfate Sodium Induced Chronic Colitis Model in Mice

Dongwoo Kim, Ja Seol Koo, Soon Ha Kim, Yeong Seo Park, Jung Wan Choe, Seung Young Kim, Jong Jin Hyun, Sung Woo Jung, Young Kul Jung and Hyung Joon Yim

Supplement Table S1. Primer sequences for real-time PCR.

Target gene		Sequences 5′ → 3′
Beta-actin	Forward	AGTGTGACGTTAACATCCGTA
	Reverse	GCCAGAGTAATCTCCTTCT
TNF-α	Forward	ACGTCGTAGCAAACCACCAA
	Reverse	TCCCTCAGGGGTGTCCTTAG
HMGB1	Forward	GGCTGACAAGGCTCGTTATG
	Reverse	CAGGATGCTCGCCTTTGATT
IL-6	Forward	CGGAGGCTTAATTACACATG

Supplement Table S2. Antibodies for Western blot assay.

Antibody	Source information	Dosage
GAPDH	Cell Cell signaling Technology (Rabbit mAb #5174)	1:5000
iNOS	BD Transduction (Mouse mAb 610431)	1:5000
NF-κB	Cell signaling Technology (Rabbit mAb #8242)	1:5000
p-NF-κB	Cell signaling Technology (Mouse mAb #13346S)	1:5000
